# Therapeutic Mechanisms of Phenothiazine Drugs: A Mini-Review of Advances in Cancer Treatment and Antibiotic Resistance

**DOI:** 10.5812/ijpr-157923

**Published:** 2025-02-08

**Authors:** Xuewei Zou, Bai Xie

**Affiliations:** 1 College of Veterinary Medicine of Yangzhou University, Yangzhou, China

**Keywords:** Phenothiazines, Therapeutic Mechanisms, Anticancer Activity, Antibiotic Resistance, Combination Therapy

## Abstract

**Context:**

Cancer and antibiotic resistance are critical global health challenges. Phenothiazines, initially developed as antipsychotics, have demonstrated diverse biological activities, including antitumor, antibacterial, and antioxidant effects. Advances in phenothiazine synthesis have produced derivatives with broader therapeutic potential by modulating receptors, ion channels, and inducing lysosomal cell death. This review explores the therapeutic potential of phenothiazines in oncology and infectious disease management, focusing on their mechanisms of action and future clinical applications.

**Evidence Acquisition:**

This narrative review synthesizes insights from relevant preclinical studies on phenothiazines' applications in oncology and infectious diseases. Mechanistic pathways and experimental outcomes were analyzed to highlight therapeutic potentials and limitations.

**Results:**

Phenothiazines demonstrate significant potential in oncology by inhibiting tumor growth via apoptosis induction, pathway modulation (e.g., PDK1/Akt, MAPK/ERK1/2, and Akt/mTOR), angiogenesis suppression through vascular endothelial growth factor (VEGF) production inhibition, and enhancing the effectiveness of monoclonal antibody therapies. They disrupt key cancer-promoting pathways and induce lysosomal cell death. Beyond oncology, phenothiazines exhibit antibacterial activity, targeting efflux pumps (Eps) and restoring antibiotic susceptibility in multidrug-resistant (MDR) pathogens. These multifaceted actions position phenothiazines as promising agents for combination therapies in cancer treatment and antibiotic-resistant infections.

**Conclusions:**

Phenothiazines hold promise as adjuvants in cancer and antibiotic resistance management. Further research should focus on optimizing their pharmacological profiles, elucidating molecular mechanisms, and validating their efficacy through clinical trials.

## 1. Context

Phenothiazines have demonstrated remarkable therapeutic versatility since their introduction in the 1950s. Initially recognized for their antipsychotic properties, particularly chlorpromazine's impact on schizophrenia treatment through D2 receptor antagonism, their applications extend to diverse conditions, including acute intermittent porphyria, methemoglobinemia, anxiety, and nausea. This broad efficacy stems from their unique chemical structure, enabling interactions with a wide range of biological targets (1).

Phenothiazines are a class of cationic and amphiphilic compounds featuring two phenyl rings and a thiazine ring containing sulfur and nitrogen, with an alkyl bridge connected to the nitrogen atom in the thiazine ring (2). The substituents at position C-2 of the tricyclic phenothiazine ring and the length of the alkyl bridge connecting the nitrogen atom at position 10 (N-10) to the terminal amine group significantly influence their anticancer efficacy (3, 4). The type of substituents in the phenothiazine ring, more than the side chain nature, plays a crucial role in their effectiveness against cancer cells (5). Modifications to the phenothiazine ring have led to derivatives such as benzo[α]phenothiazines and azaphenothiazines, which exhibit notable anticancer effects on various cell lines in vitro (6, 7).

Primarily, phenothiazines are neuroleptic medications used to treat schizophrenia and other psychiatric disorders (8). The first derivative, chlorpromazine, synthesized in the early 1950s by Rhone-Poulenc during the search for antihistaminic drugs, revolutionized psychopharmacology with its antipsychotic properties (9). Phenothiazines also serve as potent antiemetics (10). Although their exact mechanism of action is not fully elucidated, they primarily inhibit dopamine receptors in the mesolimbic pathway, particularly the D2 receptor, counteracting dopamine hyperactivity and reducing positive schizophrenia symptoms such as delusions and hallucinations (11). They also interact with adrenergic, H1 histaminergic, and serotoninergic receptors (12), modulate GABA-mediated inhibitory synaptic transmission in cultured hippocampal neurons (13), and inhibit voltage-gated Kv1.3 channels in T lymphocytes (14). However, their use is associated with side effects such as extrapyramidal symptoms and cardiac arrhythmias (15, 16), prompting the development of second-generation antipsychotics.

**Figure 1.**
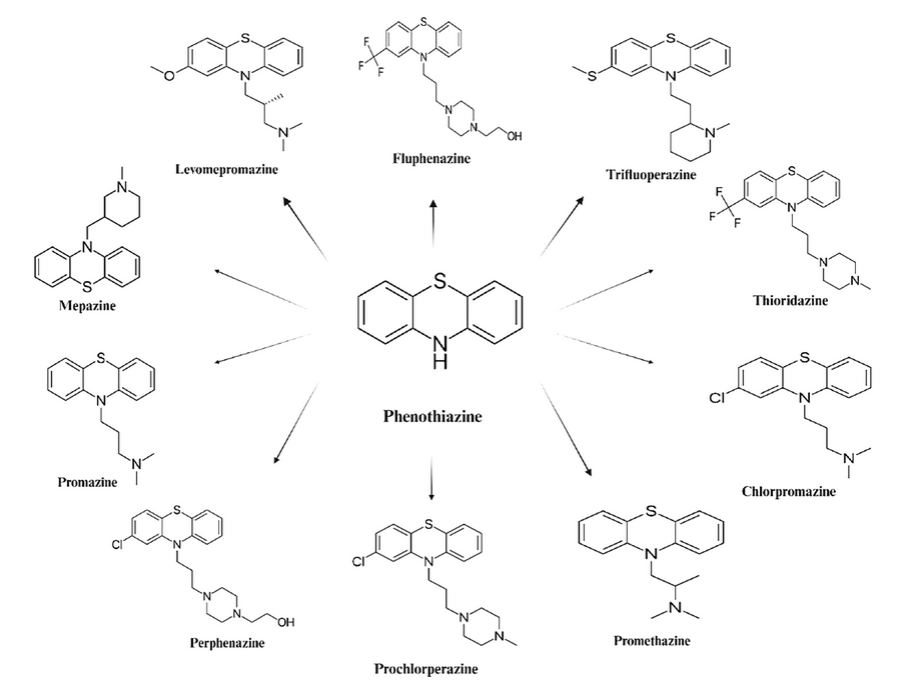
Structure of phenothiazine and well-known marketed phenothiazine derivatives with demonstrated anti-cancer effects

Structurally, phenothiazines consist of a 1,4-thiazine nucleus connected to two benzene rings (Figure 1). The substituents at positions 2 and 10 of the benzothiazine structure influence pharmacological activity (17). Based on the ligand at position 10, phenothiazines are classified as aliphatic, piperazine, or piperidine, with chlorpromazine, trifluoperazine, and thioridazine as respective examples. These substituents also affect cytotoxicity and other cellular processes. The amphiphilic nature of phenothiazines allows interaction with biological membranes, modifying their properties (18-20). Additionally, phenothiazines, as lysosomotropic compounds, accumulate in lysosomes, potentially causing lysosomal permeabilization, disruption of intracellular trafficking, and cell death (21-23). They also interfere with endocytosis; chlorpromazine and trifluoperazine inhibit dynamin- and clathrin-mediated endocytosis, enhancing the efficacy of monoclonal antibody-targeted cancer treatments by increasing protein availability for natural killer cell-mediated antibody-dependent cytotoxicity (24, 25).

Understanding the molecular mechanisms of phenothiazines' antitumor actions is crucial for their potential repurposing as adjuvants in cancer chemotherapy. Despite extensive study, further investigation into phenothiazines’ mechanisms, particularly in combination therapies for cancer and antibiotic resistance, is essential. This review examines these therapeutic potentials, focusing on phenothiazines’ applications in oncology and infectious disease.

## 2. Evidence Acquisition

### 2.1. Combination Therapies with Phenothiazines: Recent Advances

Phenothiazines, particularly chlorpromazine, thioridazine, and promethazine, exhibit cytotoxic effects on cancer cells and modulate bacterial resistance, making them promising candidates for combination therapies. Their multi-target actions—including efflux pump (EP) inhibition, membrane destabilization, and apoptosis induction—enhance the efficacy of chemotherapy and antibiotics (26). Studies indicate that phenothiazines sensitize cancer cells to conventional drugs by inhibiting EPs such as P-glycoprotein, thereby boosting intracellular drug levels, disrupting metabolic pathways, and reducing autophagy, which collectively increase apoptosis in resistant cells (27).

Thioridazine, for example, has demonstrated synergy with carboplatin, doxorubicin, and cisplatin in resistant breast and lung cancers, highlighting its potential in treating challenging tumors (28). Phenothiazines also combat multidrug-resistant (MDR) bacteria by inhibiting bacterial EPs and enhancing antibiotic efficacy. When combined with antibiotics like ampicillin and ciprofloxacin, they restore susceptibility in MDR strains by disrupting bacterial membranes—a strategy effective against *Burkholderia pseudomallei*, *Escherichia coli*, and *Staphylococcus aureus*.

Although dosage optimization remains critical to minimize toxicity, ongoing research into modified derivatives and nanoparticle delivery systems may improve the safety and precision of phenothiazine-based therapies (29).

### 2.2. Phenothiazines and Cancer: Advances in Therapeutic Mechanisms

**Figure 2.**
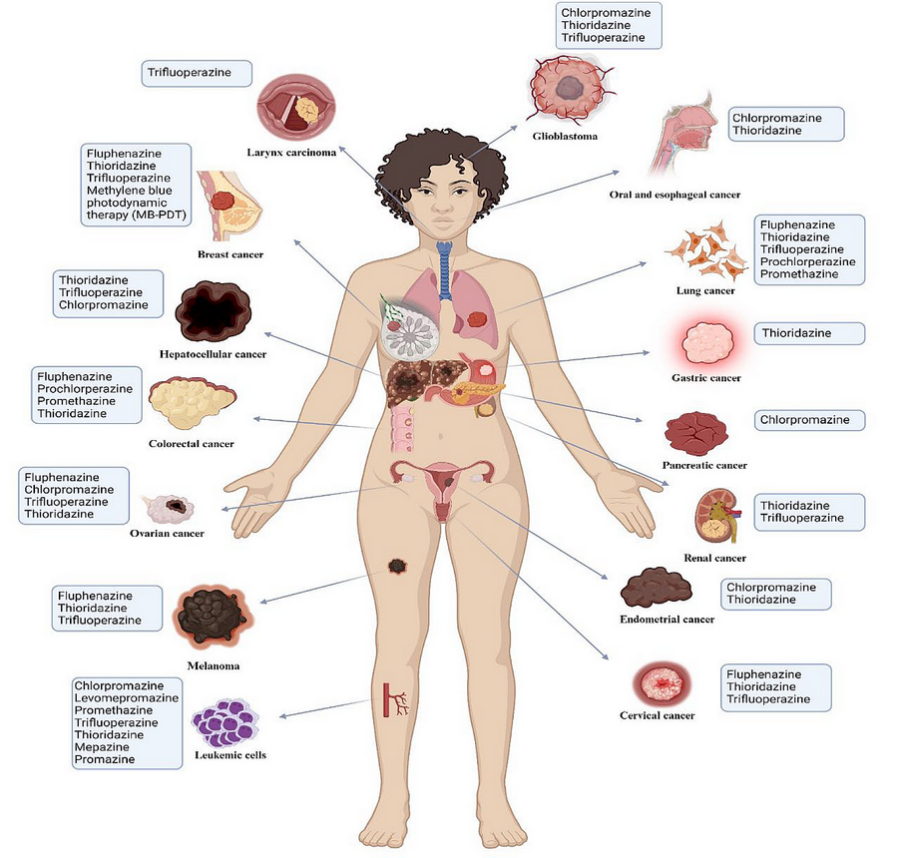
Summary of phenothiazines investigated for antitumor activity and their target malignancies. This figure offers a complete summary of different phenothiazines studied for their anti-cancer effects and the types of cancers they target. It outlines the particular cancers that these medications have shown effective anti-cancer effects against. Every phenothiazine is paired with its specific target cancer, demonstrating the variety of cancers that phenothiazines are able to treat.

Phenothiazines exhibit promising antitumor effects across various cancer types through diverse mechanisms (Figure 2 and Table 1). These compounds induce cell cycle arrest and apoptosis and target critical cancer pathways, such as PI3K/AKT, mTOR, and FOXO transcription factors, thereby disrupting tumor growth and enhancing tumor sensitivity to therapies (30, 31).

**Table 1.** Anti-tumor Activities and Mechanisms of Action of Phenothiazines Across Various Cancer Types

Phenothiazine	Cancer Types	Anti-tumor Activity	Mechanisms of Action
**Chlorpromazine**	Glioma, leukemia, endometrial, glioblastoma, pancreatic, hepatocellular carcinoma, oral cancer, hepatoma	Cytotoxic, induces apoptosis, inhibits cell proliferation	Inhibits cytochrome c oxidase in COX4-1 expressing cells, induces autophagic cell death, disrupts K-Ras membrane binding, promotes K-Ras cytoplasmic translocation, increases ROS, inhibits PI3K/AKT/mTOR, activates ER stress and UPR (ATF6-α)
**Fluphenazine**	Melanoma, colon and breast cancer, triple-negative breast cancer, ovarian carcinoma, doxorubicin-resistant colon cancer, glioblastoma	Anti-proliferative, induces apoptosis, enhances immune response, enhances drug efficacy	Induces G0/G1 arrest, mitochondrial apoptosis, DNA damage via γ-H2AX, activates caspase-3, reduces mitochondrial potential, blocks PI3K-AKT-mTOR pathways, enhances sensitivity to chemotherapy, inhibits autophagy
**Thioridazine**	Cervix, breast, NSCLC, ovarian, glioblastoma, colorectal, esophageal, hepatocellular carcinoma, leukemia, gastric cancer, larynx, renal, melanoma	Induces apoptosis, reduces viability, inhibits metastasis, enhances chemotherapy sensitivity	G0/G1 arrest, caspase activation, inhibition of PI3K/AKT/mTOR/p70S6K, mitochondrial apoptosis, enhances AMPK activity, increases ROS, reduces Bcl-2, activates autophagy, suppresses stemness genes
**Trifluoperazine**	Melanoma, triple-negative breast cancer, glioblastoma, squamous cell carcinoma, colon, osteosarcoma, hepatocellular carcinoma, larynx, renal carcinoma	Anti-proliferative, reduces viability, enhances radiation-induced cell death, mitochondrial damage	Lysosomal dysfunction, autophagy inhibition, induces G0/G1 arrest, reduces cyclin/CDK levels, interferes with Ca^2+^ signaling, activates ROS and mitophagy, enhances Bax/Bcl-2 ratio, induces apoptosis, enhances doxorubicin efficacy
**Prochlorperazine**	Squamous cell carcinoma, NSCLC, leukemia	Increases cancer cell death, boosts antibody efficacy, sensitizes to stress factors	Alters EGFR distribution, reduces Akt/mTOR signaling, binds KRAS GTP-binding pocket, activates p53, p21, γH2AX, enhances ROS, induces apoptosis through DNA damage and cell cycle arrest
**Promethazine**	Leukemia, colorectal cancer, small cell lung cancer	Cytotoxic, reduces growth, induces mitochondrial apoptosis, enhances radiation sensitivity	Activates AMPK, inhibits PI3K/AKT/mTOR, induces autophagy, triggers cell death through GPCR inhibition and JNK/c-Jun signaling, reduces cyclin levels in colorectal cancer

Abbreviations: NSCLC, non-small cell lung cancer; ROS, reactive oxygen species.

For instance, chlorpromazine has shown efficacy in glioma, endometrial cancer, and pancreatic cancer by promoting apoptosis, inhibiting cell proliferation, and blocking key signaling pathways (32, 33). It enhances autophagic cell death in glioblastoma and disrupts K-Ras binding in pancreatic cancer, promoting its cytoplasmic translocation and subsequent cell death (34). Similarly, fluphenazine is effective against melanoma, triple-negative breast cancer, and colon cancer, inducing mitochondrial apoptosis, cell cycle arrest, and disruption of the PI3K/AKT/mTOR pathways, thereby reducing tumor growth and enhancing immune responses (35, 36).

Thioridazine demonstrates potent antiproliferative effects in ovarian, lung, and cervical cancers through G0/G1 arrest, mitochondrial apoptosis, and inhibition of vascular endothelial growth factor (VEGF) and PI3K/mTOR pathways (37). Prochlorperazine enhances the efficacy of anticancer antibodies in squamous cell carcinoma by targeting EGFR distribution (25), while promethazine shows cytotoxic effects in leukemia and colorectal cancer by promoting autophagy and mitochondrial apoptosis (38, 39).

Beyond direct anticancer effects, phenothiazines modulate tumor-promoting pathways, such as tumor angiogenesis and oxidative stress. For example, they reduce VEGF production and affect angiogenesis-related signaling (40), while compounds like chlorpromazine alter oncogenic pathways in glioblastoma and endometrial cancers, impacting p53, Ras, and dopamine receptor pathways (41). Additionally, phenothiazines promote mitophagy through the AMPK/mTOR/ULK1 pathway, enhancing autophagy and mitigating mitochondrial damage in cancer cells (30). Furthermore, phenothiazines activate the Nrf2/ARE pathway, bolstering antioxidant defenses and selectively increasing cancer cell apoptosis (40). Trifluoperazine, in particular, enhances FOXO1 nuclear retention and reverses resistance mechanisms in lung adenocarcinoma by activating the KLF6/FOXO1 pathway (42).

Despite these promising preclinical results, further research is required to optimize phenothiazine derivatives for clinical application in cancer therapy, particularly to harness their broad and complex mechanisms of action across multiple tumor-promoting processes. In Table 2, we summarize studies investigating the antitumor activities of phenothiazines.

**Table 2.** Studies Investigating Anti-Tumor Activities and the Relevant Mechanisms of Phenothiazines

Authors, References	Phenothiazine	Cancer Type	Anti- Tumor Activity	Mechanisms of Action
**Oliva et al. (** 32 **)**	Chlorpromazine	Glioma (temozolomide-resistant)	Cell cycle arrest, increased survival in mice	Inhibits cytochrome c oxidase selectively in COX4-1 expressing cells
**Zhelev et al. (** 43 **)**	Chlorpromazine, levomepromazine, promethazine, trifluoperazine, thioridazine	Leukemia (various)	Anti-proliferative, cytotoxic, induces apoptosis	Enhances phosphatidylserine-annexin V complexes, triggers DNA fragmentation
**Cui et al. (** 33 **)**	Chlorpromazine	Endometrial cancer	Reduces proliferation, migration, increases apoptosis	Upregulates PRB, PI3K/AKT pathway inhibition
**Matteoni et al. (** 34 **)**	Chlorpromazine	Glioblastoma	Cytotoxic autophagy, mitotic arrest	ER stress, UPR activation via ATF6-α nuclear translocation
**Eisenberg et al. (** 44 **)**	Chlorpromazine	Pancreatic cancer (PANC-1)	Inhibits wound healing and colony formation	Disrupts K-Ras membrane binding, promoting its cytoplasmic translocation and inducing cell death
**Klutzny et al. (** 45 **)**	Fluphenazine	Colon and breast cancer	Anti-proliferative via cell cycle arrest	Inhibits acid sphingomyelinase, activates hypoxia stress pathways
**Xi et al. (** 35 **)**	Fluphenazine	Melanoma	Reduces growth, boosts immune response	G0/G1 arrest, mitochondrial apoptosis, DNA damage via γ-H2AX
**Xu et al. (** 46 **)**	Fluphenazine	Triple-negative breast cancer	Inhibits metastasis in brain and lungs	Blocks PI3K-AKT-mTOR pathways, reduces mitochondrial potential
**Heitmann et al. (** 47 **)**	Thioridazine, fluphenazine, trifluoperazine	Cervix, breast, NSCLC	Sensitizes cells to stress factors	Inhibits annexin-mediated repair, decreases membrane fluidity
**Chew et al. (** 25 **)**	Prochlorperazine	Squamous cell carcinoma	Boosts efficacy of anti-cancer antibodies	Alters EGFR distribution, reduces Akt/mTOR signaling
**Sad et al. (** 48 **)**	Prochlorperazine	NSCLC	Increases cancer cell death and survival rates in mice	Binds KRAS GTP-binding pocket, keeping mutant K-Ras in inactive form; with radiation, activates p-ATM, p53, p21, and γH2AX, promoting cell cycle arrest and apoptosis
**Medeiros et al. (** 38 **)**	Promethazine	Leukemia	Cytotoxic via autophagy	Activates AMPK, inhibits PI3K/AKT/mTOR pathway
**Tan et al. (** 39 **)**	Promethazine	Colorectal cancer	Suppresses growth, induces mitochondrial apoptosis	PI3K/AKT pathway inhibition
**Jahchan et al. (** 49 **)**	Promethazine	Small cell lung cancer	Reduces growth, triggers cell death	Inhibits GPCRs, activates JNK/c-Jun signaling
**Kang et al. (** 50 **)**	Thioridazine	Cervical, endometrial	Apoptosis induction	G1 arrest, PI3K/Akt/mTOR/p70S6K inhibition
**Park et al. (** 51 **)**	Thioridazine	Ovarian cancer	Reduces angiogenesis	Blocks VEGFR-2/PI3K/mTOR pathway
**Song et al. (** 52 **)**	Thioridazine	Triple-negative breast cancer	Inhibits growth and migration	PI3K/Akt/mTOR/p70S6K inhibition, G1 arrest
**Cheng et al. (** 53 **)**	Thioridazine	Glioblastoma	Autophagy induction	Enhances AMPK activity, regulates VEGFR-2
**Gil-Ad et al. (** 54 **)**	Thioridazine	Melanoma	Potent anti-proliferative effects	DNA fragmentation, caspase-3 upregulation
**Zhang et al. (** 55 **)**	Trifluoperazine	Melanoma	Reduces viability, extends survival in mice	Lysosomal damage, autophagic flux inhibition
**Feng et al. (** 56 **)**	Trifluoperazine	Triple-negative breast cancer	Inhibits cell proliferation	Reduces cyclinD1/CDK4, cyclin E/CDK2 levels
**Zhang et al. (** 57 **)**	Trifluoperazine	Glioblastoma	Enhances sensitivity to radiotherapy	Inhibits autophagy, reduces DNA repair proteins
**Choi et al. (** 58 **)**	Fluphenazine, chlorpromazine, trifluoperazine, thioridazine	Ovarian carcinoma	Inhibits tumor growth	Reduces Akt phosphorylation, suppresses PDK1 kinase activity
**Kang et al. (** 59 **)**	Trifluoperazine	Glioblastoma	Time- and dose-dependent cytotoxicity, blocks growth and spread	Induces sustained Ca²⁺ release via IP3R by binding CaM2, enhancing responsiveness in glioblastoma cells
**Sroda-Pomianek et al. (** 36 **)**	Fluphenazine	Doxorubicin-resistant colon cancer	Enhances doxorubicin efficacy	Lowers ABCB1 and COX-2 expression, increases Bax/Bcl-2 ratio for apoptosis
**Gangopadhyay et al. (** 60 **)**	Trifluoperazine	Larynx, melanoma	Enhances radiation-induced death	Interferes with Ca^2+^ signaling, induces apoptosis
**Colturato-Kido et al. (** 61 **)**	Thioridazine	Acute lymphoblastic leukemia	Programmed cell death induction	Increases NOXA/MCL-1 ratio, AMPK/PI3K/AKT/mTOR inhibition
**Li et al. (** 62 **)**	Thioridazine	Esophageal carcinoma	Reduces viability with radiation	G0/G1 arrest, caspase activation, Bcl-2 downregulation
**Shen et al. (** 63 **)**	Thioridazine	Lung, ovary	Enhances cisplatin chemotherapy	Mitochondrial apoptosis, reduces Bcl-2
**El-Sayed Ibrahim et al. (** 40 **)**	Thioridazine	Hepatocellular carcinoma (HepG2)	Reduces cell proliferation and increases ROS	Downregulates PI3K/AKT and SIRT1/NRF2 expression, lowers VEGF levels, raises oxidative stress
**Min et al. (** 64 **)**	Thioridazine	Renal carcinoma, breast carcinoma, glioma	Induces apoptosis, enhances TRAIL sensitivity	Decreases c-FLIP(L) and Mcl-1 via proteasome activity, suppresses Akt pathway, increases ROS in renal carcinoma cells
**Qian et al. (** 37 **)**	Thioridazine	Human lung and ovary cancers	Enhances cisplatin efficacy, induces apoptosis	Causes mitochondrial-dependent apoptosis via G0/G1 arrest, activates caspase 9, increases Bax, decreases Bcl-2
**Seervi et al. (** 65 **)**	Thioridazine	Cervical, fibroblast	Bax-Bak dependent apoptosis	ROS increase, ER stress induc
**Zhang et al. (** 66 **)**	Thioridazine	Colorectal cancer	Reduces growth and spread of CSCs	Increases Bax, caspase-3, decreases Bcl-2
**Mu et al. (** 67 **)**	Thioridazine	Gastric cancer	Cytotoxicity and colony suppression	Caspase-dependent apoptosis, mitochondrial pathway activatio
**Chen et al. (** 68 **)**	Thioridazine	Hepatocellular carcinoma	Induces Ca^2+^-independent cell death	Activates Ca^2+^ signaling via PKC-responsive pathways
**Moraes et al. (** 69 **)**	Thioridazine	Leukemia	Selective leukemia cell apoptosis	Increases cytosolic Ca^2+^, caspase 9/3 activation, ER stress
**Shin et al. (** 70 **)**	Chlorpromazine	Glioma	Inhibits growth, colony survival	Autophagic cell death, PI3K/AKT/mTOR inhibition
**Jhou et al. (** 41 **)**	Chlorpromazine	Oral cancer	Growth suppression, apoptosis	Death receptor and mitochondrial pathway activation
**Goyette et al. (** 71 **)**	Thioridazine, fluphenazine, trifluoperazine	Triple-negative breast cancer	Reduces invasion, proliferation, increases apoptosis	Decreases PI3K/AKT/mTOR and ERK s
**Seo et al. (** 72 **, ** 73 **)**	Thioridazine, curcumin	Head, neck, breast, glioma	Induces cell death in combination	Boosts PSMA5 expression, decreases c-FLIP, Mcl-1
**Nagel et al. (** 74 **)**	Mepazine, thioridazine, promazine	Diffuse large B cell lymphoma	Selective cytotoxicity in ABC-DLBCL	Inhibits MALT1, reduces viabilit
**Lu et al. (** 75 **)**	Thioridazine	Hepatocellular carcinoma	Decreases viability, migration	G0/G1 arrest, stemness gene inhibitio
**Spengler et al. (** 76 **)**	Thioridazine	MDR lymphoma	Promotes apoptosis	ABCB1 transporter inhibition
**Antherieu et al. (** 77 **)**	Chlorpromazine	Hepatoma	Induces oxidative stress	Alters bile acid transport and CYP8B1 expression
**Yue et al. (** 78 **)**	Thioridazine	Lung cancer stem cells	Reduces sphere formation	Decreases Akt phosphorylation, affects stem cell propertie
**Dos Santos et al. (** 79 **)**	Methylene blue PDT	Breast adenocarcinoma	Significant cancer cell destruction	Oxidative damage, autophagy induction
**Harris et al. (** 80 **)**	Chlorpromazine	Hepatocellular carcinoma	Decreases cell viability, increases ROS	Early apoptosis induction
**Shen et al. (** 30 **)**	Trifluoperazine	Osteosarcoma	Suppresses proliferation	ROS accumulation, mitochondrial damage, mitophagy induction
**Jiang et al. (** 42 **)**	Trifluoperazine	Hepatocellular carcinoma	Reduces viability, enhances apoptosis	Enhances Bax/Bcl-2 ratio, FOXO1 nuclear localization

Abbreviations: NSCLC, non-small cell lung cancer; VEGF, vascular endothelial growth factor; ROS, reactive oxygen species; PSMA5, proteasome subunit alpha 5; COX-2, cyclooxygenase-2; ABC, ATP-binding cassette.

### 2.3. Phenothiazines Induce Cell Cycle Arrest and Apoptosis in Cancer Cells

Phenothiazines effectively induce cell cycle arrest and apoptosis across diverse cancer types. Fluphenazine and trifluoperazine, for instance, suppress metastasis in melanoma by causing G0/G1 phase arrest and initiating mitochondria-mediated apoptosis. In melanoma, the cyclin D–CDK4/6 pathway, frequently overactivated in metastatic cases, drives G1-S phase transition and cellular proliferation, while the RAS/RAF/MEK/MAPK pathway further amplifies cyclin D1 expression, sustaining this cycle (35, 81, 82). Trifluoperazine disrupts these processes by downregulating cyclins D and E, CDK2, CDK4, and c-Myc, while upregulating p21 and p27, leading to G0/G1 arrest and diminished tumor growth. Similarly, in hepatocellular carcinoma (HCC), trifluoperazine induces G0/G1 arrest and apoptosis by increasing the Bax/Bcl-2 ratio, effectively reducing cell viability (42, 81).

Moreover, novel phenothiazine derivatives demonstrate antiproliferative effects in lung and pancreatic cancer cell lines by inducing G0/G1 phase arrest and reducing S and G2/M phase proportions, highlighting their ability to impair cell cycle progression and promote apoptosis. These findings underscore the potential of phenothiazines as therapeutic agents targeting cell cycle dysregulation in cancer (83).

### 2.4. Phenothiazines Modulate Forkhead Box O Factors to Induce Cell Cycle Arrest and Apoptosis

Forkhead box O (FOXO) proteins, including FOXO1, FOXO3, FOXO4, and FOXO6, function as tumor suppressors by regulating cell cycle arrest, DNA repair, and apoptosis through pathways involving cyclins, Bcl-6, Fas ligand (FasL), and TRAIL (84). Trifluoperazine, a phenothiazine derivative, enhances FOXO1 nuclear retention and counters AKT-driven resistance to erlotinib by activating the KLF6/FOXO1 signaling pathway in lung adenocarcinoma. This is linked to its inhibition of calmodulin, which facilitates FOXO1 nuclear relocalization (85). In hepatocellular carcinoma, trifluoperazine increases FOXO1 nuclear expression, elevating Bax levels and reducing Bcl-2, thereby raising the Bax/Bcl-2 ratio to promote apoptosis. Similarly, AKT inhibition triggers FOXO3a nuclear translocation and acetylation, forming a transcriptional complex that upregulates CDK6, contributing to intrinsic resistance to AKT inhibitors (AKTi) (42). Targeting the FOXO3a-BRD4 complex or CDK6 enhances AKTi's antiproliferative and pro-apoptotic effects. These findings highlight phenothiazines’ potential to modulate FOXO factors and support future research into combination therapies with AKTi (31).

### 2.5. Phenothiazines Modulate Key Signaling Pathways in Cancer Cells

Phenothiazines disrupt critical cancer signaling pathways, including PI3K/Akt/mTOR and MAPK/ERK1/2, to inhibit proliferation, induce apoptosis, and enhance autophagy. Chlorpromazine, for instance, exhibits antitumor activity by modulating p53, Ras, and dopamine receptor D2 (DRD2), which regulate cell cycle progression, metastasis, and chemoresistance. In endometrial cancer, it downregulates IGF-IR and PI3K/Akt phosphorylation via DRD2, while in glioblastoma, it disrupts K-Ras membrane localization, leading to apoptosis and autophagy. Chlorpromazine also induces ER stress and oxidative damage, selectively targeting chemoresistant cells (34, 61, 80, 86).

Thioridazine further modulates pathways like PI3K/Akt/mTOR, suppressing tumor growth by downregulating cyclins and upregulating p21 and p27. It enhances ER stress via the eIF2α/ATF4/CHOP pathway, promoting immunogenic cell death and synergizing with oxaliplatin in colorectal cancer models. In glioblastoma, thioridazine activates AMPK and Wnt/β-catenin signaling, inducing autophagy-associated apoptosis and sensitizing cells to temozolomide by blocking autophagic flux (50, 87-89). These findings highlight phenothiazines’ potential to target diverse pathways and overcome resistance mechanisms in cancer therapy.

### 2.6. Phenothiazines Inhibit Tumor Angiogenesis

Phenothiazines inhibit angiogenesis—essential for tumor growth—by suppressing VEGF production and interfering with VEGF-mediated signaling, along with other angiogenesis-related pathways like MAPK (26). Trifluoperazine treatment has been shown to reduce microvessel density (MVD) and VEGF levels in vivo, thereby limiting tumor growth (42). Pulkoski-Gross et al. (2015) found that trifluoperazine notably decreased angiogenesis, reduced VEGF expression, and inhibited cancer cell invasion by lowering phosphorylated AKT and β-catenin levels (90). Additionally, thioridazine disrupts pathways downstream of PI3K, including Akt, PDK1, and mTOR, through VEGFR-2, thereby modulating endothelial cell function and inhibiting angiogenesis. This suggests thioridazine’s potential as an anti-angiogenic agent, particularly in ovarian cancer (91).

### 2.7. Phenothiazines and the AMPK/mTOR/ULK1 Pathway in Cancer Mitophagy

Mitophagy, a selective form of autophagy, removes and recycles damaged mitochondria. In cancer, reduced mitophagy can promote tumor growth by allowing damaged mitochondria to accumulate, increasing oxidative stress and genomic instability (92). Reactive oxygen species (ROS) can activate AMPK, a key regulator of autophagy and energy metabolism, while mTOR negatively regulates autophagy when phosphorylated, and ULK1 initiates autophagy (93). Research has shown that mitophagy regulation involves the AMPK/mTOR/ULK1 pathway (94). Recent studies highlight the role of this pathway in malignancy (95). Shen et al. (2024) found that trifluoperazine exerts anti-osteosarcoma effects by enhancing mitophagy through the AMPK/mTOR/ULK1 pathway. Trifluoperazine reduced P62 and increased LC3B II, ATG5, and Bclin-1 expression, markers of autophagy in osteosarcoma cells (30). It also promoted mitochondrial damage through ROS accumulation, thereby inducing mitophagy and displaying antioxidant effects (30).

### 2.8. Phenothiazines and Nrf2-Mediated Pathways in Oxidative Stress Modulation

Phenothiazines modulate oxidative stress, a condition characterized by excessive reactive oxygen and nitrogen species (ROS/RNS) that damage proteins, nucleic acids, and lipids, contributing to inflammation, apoptosis, and cancer progression. These compounds activate the Nrf2/ARE pathway, which enhances antioxidant gene expression and suppresses pro-inflammatory mediators like iNOS. In cancer, Nrf2 acts as a double-edged sword, suppressing tumor growth while also promoting drug resistance under stress (96-98). Thioridazine demonstrates Nrf2 activation by increasing ROS production, reducing SIRT1/Nrf2 expression, and inhibiting proliferation in liver cancer cells. It also upregulates proteasome subunit alpha 5 (PSMA5) and, in combination with curcumin, enhances Nrf2 activity via NOX4-dependent ROS, leading to proteasomal degradation of anti-apoptotic proteins like C-FLIP and Mcl-1. These actions highlight phenothiazines' potential in targeting oxidative stress pathways (40, 72). While promising preclinical results suggest therapeutic potential for oxidative stress-linked diseases, including cancer, no phenothiazine derivatives are clinically approved for Nrf2 modulation. Further research is needed to optimize these agents for clinical application.

### 2.9. Phenothiazines and Cyclooxygenase-2 Inhibitory Activity as a Possible Mechanism for Anticancer Activity

Cyclooxygenase-2 (COX-2) contributes to carcinogenesis and the malignant characteristics of cancer cells through at least six different pathways, including immunological regulation, increased mutagenesis, improved invasion, increased angiogenesis, and suppression of apoptosis (99). Tumor development is often accompanied by inflammation, which is associated with increased expression of COX-2, the inducible form of cyclooxygenase typically absent in healthy tissue (100, 101). Additionally, higher COX-2 levels have been observed in MDR cells compared to chemotherapy-sensitive cancer cells (102, 103).

Although concerns about the potential toxic effects of systemic selective inhibition have raised doubts about the safety of COX-2 inhibition as a chemotherapy prevention technique, selective COX-2 inhibitors were initially considered promising candidate chemotherapy drugs (99). Some phenothiazine derivatives have been reported to possess anti-inflammatory activity, though most reports originate from animal studies and do not discuss the molecular mechanism of their anti-inflammatory action (104, 105). An approach based on computational and combinatorial chemistry methods yielded a phenothiazine-type lead compound that was a selective COX-2 inhibitor (106).

Prior to 2019, the impact of phenothiazine derivatives on COX-2 was poorly understood. However, Sroda-Pomianek et al. (2019) investigated the effects of fluphenazine, MAE-TPR, and APh-FLU, both individually and in combination with simvastatin, on cell growth, apoptosis, and COX-2 activity and expression within the LoVo/Dx cell line (36). Simvastatin and all phenothiazine derivatives, except APh-FLU, reduced the expression of COX-2 protein. The combined treatment of LoVo/Dx cells with phenothiazine derivatives and simvastatin resulted in a further decrease in COX-2 activity. However, the reduction of COX-2 protein expression was similar in both single-agent treatment and the application of the phenothiazine derivatives: Simvastatin mixture (36).

## 3. Results

### 3.1. Phenothiazines: Antibacterial Effects and Antibiotic Resistance

Antimicrobial resistance (AMR) poses a critical threat to global health, with the World Health Organization (WHO) identifying it as a "silent pandemic" and one of the top ten global health threats. Antimicrobial resistance is driven by factors such as bacterial evolution, antibiotic overuse, and inadequate public awareness, and it is projected to cause up to 10 million deaths annually by 2050 (107). To address this urgent threat, the WHO has prioritized certain pathogens, including *Mycobacterium tuberculosis*, which causes 1.8 million deaths each year, and has classified pathogens into critical (e.g., carbapenem-resistant *Acinetobacter baumannii*, *Pseudomonas aeruginosa*), high (e.g., vancomycin-resistant *Enterococcus faecium*, MRSA), and medium priority groups (e.g., penicillin-resistant *Streptococcus pneumoniae*) based on their resistance profiles (108).

The emergence of MDR and extensively drug-resistant (XDR) pathogens has outpaced the efficacy of conventional antibiotics, necessitating innovative approaches to combat AMR (109). MDR bacteria are increasingly resilient due to widespread antibiotic misuse, while MDR cancer cells present parallel challenges in cancer therapy, often leading to relapses (110). Both bacteria and cancer cells share resistance mechanisms, including drug inactivation, EP overexpression, and target modifications (111). In bacteria, EPs facilitate biofilm formation and virulence, while in cancer cells, EPs contribute to drug extrusion and metastasis (112).

### 3.2. The Antimicrobial Action of Phenothiazines

Phenothiazines, including the FDA-approved chlorpromazine, display antibacterial and EP inhibitory properties against various pathogens, such as Mycobacteria, gram-positive, and gram-negative bacteria like *P. aeruginosa*. These properties suggest their potential as adjunctive therapies to antibiotics (29). Chlorpromazine also inhibits quorum sensing in *Chromobacterium violaceum* and *Serratia marcescens* (113), while miconazole can reduce virulence gene expression in *P. aeruginosa* (114).

In vitro screening (Table 3) shows that chlorpromazine, promazine, methdilazine, fluphenazine, trimeprazine, trifluoperazine, and flupenthixol exhibit minimum inhibitory concentration (MIC) values around 10 µg/mL against most gram-positive bacteria, with methdilazine and fluphenazine being effective at concentrations as low as 2 - 5 µg/mL for some strains (115). Trifluoperazine was particularly active against gram-positive bacteria, with MICs as low as 2 µg/mL. Among gram-negative bacteria, Vibrio species were most sensitive, while Salmonella and Shigella strains showed varying sensitivities. *Klebsiella*, pseudomonads, and *Acinetobacter* species exhibited high resistance to these compounds. Most phenothiazines were bacteriostatic, though some could kill pathogens within 6 to 18 hours (115).

**Table 3.** Antibacterial Activity of Synthetic Phenothiazines by In Vitro Screening

Phenothiazine	Antibacterial Activity	MIC µg/mL for Gram Positive Organisms	MIC µg/mL for Gram Negative Organisms	Reference
**Chlorpromazine**	Bactericidal for gram positive organisms; Bacteriostatic for gram negative organisms	10 - 50	25 - 100	Dastidar et al. (115)
**Promazine**	Bacteriostatic	10 - 50	10 - 100	
**Prochlorperazine**	Bacteriostatic	25 - 100	50 - 400	
**Fluphenazine**	Bactericidal	10 - 100	10 - 100	
**Trifluoperazine**	Bactericidal	10 - 100	25 - 200	
**Thioridazine**	Bactericidal for gram positive organisms; Bacteriostatic for gram negative organisms	32 - 64 and 50 - 800	100 - 800	
**Triflupromazine**	Bactericidal	2 - 50	2 - 100	
**Flupenthixol**	Bacteriostatic	5 - 50	10 - 100	
**Promethazine**	Bacteriostatic	50 - 200	100 - 200	
**Methdilazine**	Bactericidal	10 - 100	25 - 200	
**Trimeprazine**	Bactericidal	10 - 100	10 - 100	

Abbreviation: MIC, minimum inhibitory concentration.

Table 4 summarizes studies investigating the preventative effects of phenothiazines against antibiotic resistance and their associated mechanisms.

**Table 4.** Phenothiazines Preventive Effect Against Antibiotic Resistance and the Relevant Mechanisms

Authors, References	Phenothiazine	Infection and Strains	Effect and Mechanism of Action
**Kaatz et al. (** 116 **)**	Chlorpromazine, fluphenazine, thioridazine, prochlorperazine	*Staphylococcus aureus *(various strains)	Potent inhibitors of NorA EP, especially against strains SA-K1748 and SA-K2068; increases ethidium bromide (EtBr) accumulation
**Kristiansen et al. (** 117 **)**	Chlorpromazine, thioridazine	MRSA, MSSA	Kills *S. aureus* strains irrespective of oxacillin resistance; likely EP inhibition
**Chan et al. (** 118 **)**	Prochlorperazine, chlorpromazine, promazine	*Burkholderia pseudomallei*	Enhances efficacy of aminoglycosides and macrolides by inhibiting EPs BpeAB-OprB and AmrAB-OprA, disrupting proton gradient
**Bailey et al. (** 119 **)**	Thioridazine, trifluoperazine, chlorpromazine	*Salmonella enterica* (Typhimurium)	Increases antibiotic efficacy by inhibiting AcrB EP, enhancing EtBr uptake
**Rodrigues et al. (** 120 **)**	Thioridazine, chlorpromazine	*Mycobacterium avium, Mycobacterium smegmatis*	Increases erythromycin susceptibility by inhibiting efflux, enhancing EtBr accumulation in a temperature-dependent manner
**Dutta et al. (** 121 **)**	Thioridazine	*M. tuberculosis*	Alters gene expression related to cell-envelope integrity, EPs, and stress response, inducing cell-envelope damage
**Grimsey et al. (** 122 **)**	Chlorpromazine	*Salmonella typhimurium, Escherichia coli*	Inhibits AcrB EP by binding within the hydrophobic trap, preventing substrate binding and efflux
**McCusker et al. (** 123 **)**	Thioridazine, chlorpromazine	*Enterobacter aerogenes* (various isolates)	Inhibits MDR phenotype by reducing ciprofloxacin and chloramphenicol resistance, increasing Hoechst dye accumulation
**Nove et al. (** 124 **)**	Promethazine	*E. coli* (K-12 AG100)	Induces stress response in acidic pH; upregulates EP genes (acrA, acrB) for toxic substance removal
**Sidrim et al. (** 125 **)**	Promethazine	*B. pseudomallei* (various isolates)	Disrupts biofilm structure, lowering MICs and MBECs for several antibiotics, improving antibiotic efficacy

Abbreviations: EP, efflux pump; MIC, minimum inhibitory concentration.

### 3.3. Possible Mechanisms of the Antimicrobial Action of Phenothiazines

Phenothiazines disrupt microbial growth by targeting bacterial membranes, nucleic acids, and EP mechanisms. These compounds inhibit calmodulin-like proteins in bacteria, intercalate into DNA, impair replication processes, and enhance bacterial susceptibility to antibiotics by affecting efflux systems (29, 126).

### 3.4. Phenothiazines: Targeting Bacterial Membranes and Cell Wall

Phenothiazines, such as chlorpromazine, exhibit both bacteriostatic and bactericidal effects depending on the concentration. They impair potassium transport, alter membrane permeability, and disrupt bacterial haemolysins. Chlorpromazine induces elongation and filamentation in *E. coli*, alters protein patterns in bacterial envelopes, and affects antibody recognition of the O antigen in Salmonella by binding to a 55 kDa protein (127-130). Thioridazine rapidly destroys gram-positive bacteria and damages the nuclear and cytoplasmic membranes of *Trypanosoma brucei* while accumulating in macrophages, where it reaches bactericidal levels (131, 132).

### 3.5. Phenothiazines: Targeting Nucleic Acids and Cellular Replication

Phenothiazines intercalate into DNA, leading to single-stranded breaks and disrupting essential processes like replication and transcription. Their binding affinity depends on guanine-cytosine content, and they may act as plasmid-curing agents by inhibiting plasmid replication at sub-inhibitory levels (133, 134). As calmodulin antagonists, phenothiazines disrupt calcium signaling pathways in prokaryotic and eukaryotic cells, slowing replication and cell cycle progression. For example, chlorpromazine inhibits conformational changes in *Schistosoma mansoni calmodulins* and reduces nuclear calmodulin activity in *Candida albicans*, delaying entry into the S and G1 phases (3, 135). Phenothiazines also induce morphological changes at sub-MIC levels. In *E. coli*, they cause temporary filamentation, while in *S. aureus*, they inhibit cell division and form mesosome-like structures. In *Salmonella typhimurium*, chlorpromazine alters the cell wall of resistant mutants by eliminating the 55 kDa protein, unblocking anti-O antibody binding to O antigens and enhancing antimicrobial activity (130).

### 3.6. Phenothiazines and Bacterial Efflux Pumps: Vital Pathway in Drug Resistance

Bacterial EPs expel toxins, aid stress adaptation, and contribute to virulence and drug resistance. EPs are categorized into six families: ATP-binding cassette (ABC), multidrug and toxic compound extrusion (MATE), small multidrug resistance (SMR), major facilitator superfamily (MFS), resistance-nodulation-division (RND), and proteobacterial antimicrobial compound efflux (PACE), each playing distinct roles in resistance (136). Inhibiting EPs increases bacterial susceptibility to antibiotics by raising intracellular drug concentrations. Phenothiazines act as EP inhibitors (EPIs), with pH-dependent activity, effectively targeting EPs at neutral or alkaline pH by disrupting the proton motive force (PMF)—a key energy source for efflux (137, 138).

Phenothiazines, including thioridazine, chlorpromazine, and fluphenazine, inhibit efflux systems across various bacterial species. For instance, they block NorA-mediated efflux in *S. aureus*, enhancing oxacillin susceptibility, and inhibit the proton gradient in *B. pseudomallei*, disrupting erythromycin efflux (117, 118). In *M. smegmatis* and *M. avium*, they increase intracellular ethidium bromide retention, and in *M. tuberculosis*, chlorpromazine impairs respiration and inhibits the AcrB pump in *S. typhimurium* (121). Chlorpromazine also reverses chloramphenicol resistance in *Enterobacter aerogenes*, significantly reducing its MIC (123).

Promethazine demonstrates EPI activity with notable variability across pH levels. In *E. coli* K12 AG100, its efficacy declines under acidic conditions due to the enhanced efficiency of the AcrAB-TolC pump but upregulates acrA and acrB genes as a bacterial defense mechanism (124). Promethazine also disrupts bacterial biofilms, enhancing antibiotic penetration and efficacy in *B. pseudomallei* against drugs like erythromycin and ciprofloxacin, offering a potential strategy for combating biofilm-associated resistance (125).

## 4. Conclusions

Phenothiazines represent a versatile class of compounds with significant potential beyond their psychiatric origins. This review highlights their multifaceted therapeutic applications, particularly in oncology and infectious disease. In cancer, phenothiazines induce apoptosis, disrupt critical signaling pathways, and inhibit angiogenesis. Their activation of the Nrf2/ARE pathway suggests anti-inflammatory and antioxidant potential. In infectious disease, their ability to modulate bacterial EPs underscores their role in combating antibiotic resistance and enhancing antimicrobial efficacy. These findings establish a strong foundation for the continued exploration of phenothiazines as adjunct therapies in cancer and resistant infections. Advancing our understanding of their mechanisms and optimizing their application could unlock innovative treatment options across diverse medical contexts.

### 4.1. Future Prospects and Recommendations

Originally developed as antipsychotics, phenothiazines have emerged as promising agents in oncology and infectious disease treatment. Their ability to disrupt cancer pathways—such as PDK1/Akt, MAPK/ERK1/2, and Akt/mTOR—alongside their role in apoptosis induction and angiogenesis inhibition, highlights their therapeutic versatility. Furthermore, phenothiazines’ modulation of bacterial EPs offers potential for addressing antibiotic resistance.

Future research should prioritize optimizing phenothiazine derivatives, exploring synergistic effects with existing anticancer and antimicrobial agents, and conducting robust clinical trials to validate their safety and efficacy. Investigations into their interactions with neurotransmitter systems could also reveal novel therapeutic applications. Combining phenothiazines with immunotherapies may enhance anti-tumor responses, while tailored combinations against specific cancers or bacterial pathogens could improve treatment precision.

Comprehensive pharmacokinetic studies are essential to assess long-term effects, drug interactions, and dosing strategies. Additionally, developing phenothiazine derivatives with enhanced selectivity and bioavailability could expand their therapeutic reach and improve clinical outcomes.
